# V1 neurons respond to luminance changes faster than contrast changes

**DOI:** 10.1038/srep17173

**Published:** 2015-12-04

**Authors:** Wen-Liang Wang, Ran Li, Jian Ding, Louis Tao, Da-Peng Li, Yi Wang

**Affiliations:** 1 State Key Laboratory of Brain and Cognitive Sciences, Institute of Biophysics, Chinese Academy of Sciences, Beijing 100101, China; 2 University of Chinese Academy of Sciences, Beijing 100101, China; 3 School of Optometry, University of California, Berkeley, CA 94720, USA; 4 Center for Bioinformatics, National Laboratory of Protein Engineering and Plant Genetics Engineering, College of Life Sciences, Peking University, Number 5 Summer Palace Road, Beijing 100871, China; 5 Center for Quantitative Biology, Peking University, Number 5 Summer Palace Road, Beijing 100871, China

## Abstract

Luminance and contrast are two major attributes of objects in the visual scene. Luminance and contrast information received by visual neurons are often updated simultaneously. We examined the temporal response properties of neurons in the primary visual cortex (V1) to stimuli whose luminance and contrast were simultaneously changed by 50 Hz. We found that response tuning to luminance changes precedes tuning to contrast changes in V1. For most V1 neurons, the onset time of response tuning to luminance changes was shorter than that to contrast changes. Most neurons carried luminance information in the early response stage, while all neurons carried both contrast and luminance information in the late response stage. The early luminance response suggests that cortical processing for luminance is not as slow as previously thought.

Luminance and contrast provide information regarding the surface properties of objects, from which we perceive the coarse and fine forms of objects in the visual scene. Local luminance and contrast in a given visual scene typically vary by over a factor of more than ten^1^. A neuron in the primary visual cortex (V1) receives information from a local region of the visual field, that is, its receptive field (RF). The visual information received by the eyes varies rapidly as the images on the retina change with body, head, and eye movements during natural viewing. Therefore, the luminance and contrast information within the RF of an individual V1 neuron dynamically change with time. The response properties of V1 neurons to contrast have been studied intensively. In the RFs of V1 neurons, the elongated arrangement of ON and OFF inputs from the lateral geniculate nucleus (LGN) generates the selective responses of V1 neurons to oriented contrast^2^,^3^,^4^. Stimuli with higher contrast evoke faster and stronger responses in V1 neurons than those with lower contrast^5^,^6^,^7^,^8^,^9^. Luminance changes in uniform stimuli evoke relatively weak responses in V1 neurons in comparison to contrast changes in stimuli^10^. Some V1 neurons respond to uniform luminance stimuli more slowly than to oriented contrast stimuli^11^. The responses of V1 neurons to luminance are thought to be slower than to contrast and to be generated by responses to contrast borders through the neural process of slow, scale-dependent filling-in^11^,^12^. Other studies have shown that the responses of V1 neurons carry much of the luminance information when the luminance of the stimuli varies rapidly^13^,^14^.

Under normal viewing conditions, the luminance and contrast signals that are transmitted to individual V1 neurons may change rapidly and concurrently. However, almost all previous studies have focused on V1 responses to either contrast changes or luminance changes, that is, changing the stimulus contrast when holding the stimulus luminance constant or vice versa^5^,^6^,^8^,^9^,^10^,^12^,^15^,^16^,^17^. Only a preliminary study explored the responses of V1 neurons to simultaneously changing luminance and contrast, and that study suggested that their responses are strongly modulated by both the local mean luminance and contrast^13^. Furthermore, the luminance and contrast responses of V1 neurons are largely independent^10^,^13^. This independence is consistent with the statistical independence of local luminance and contrast in natural scenes^1^,^18^. However, the dynamics of V1 responses to simultaneously changing luminance and contrast remain unclear. In the current study, we investigated the temporal properties of V1 responses to simultaneously changing luminance and contrast stimuli that were presented for 20 ms without an interval blank. Our results suggest that V1 neurons begin to tune to luminance changes earlier than they tune to contrast changes when visual information is changing rapidly.

## Results

**Figure 1:**
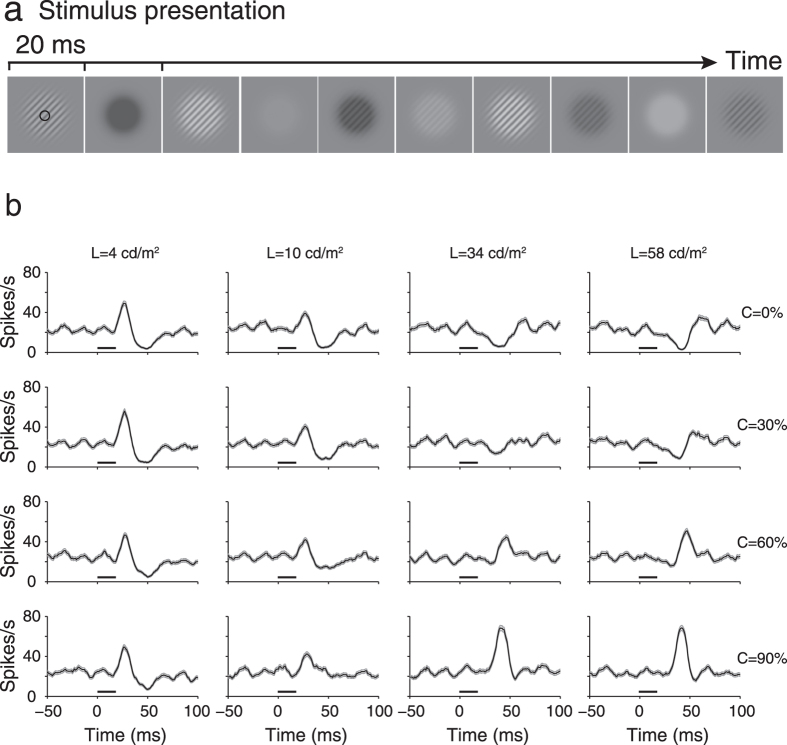
Visual stimulation and responses of V1 neurons to luminance changes and contrast changes. (**a**) Stimulus presentation. Each stimulus was presented for 20 ms (50 Hz), and all stimuli were presented randomly and consecutively. The dark, small ring inside the left-most panel represents the RF of a visual neuron in V1. (**b**) Examples of PSTHs. The PSTHs were calculated with a 1 ms bin and smoothed using a window of 11 ms. The 4 × 4 matrix of panels show PSTHs of the neuron in Fig. 2a to partial combinations of stimulus parameters (luminance: 4, 10, 34, and 58 cd/m^2^ and contrast: 0%, 30%, 60%, and 90% of a 10 × 10 matrix of stimuli with luminance at 10 levels (4–58 cd/m^2^ in 6 cd/m^2^ steps) and contrast at 10 levels (0–90% in 10% steps)). Each panel shows responses (spike rates) to a stimulus from 50 ms prior to the stimulus onset until 100 ms after the onset. The dark horizontal bar (=20 ms) indicates the duration of stimulus presentation. Shadows indicate the s.e.m. The background luminance of the CRT monitor screen was 31 cd/m^2^, which was the mean luminance of all stimuli.

A series of sinusoidal gratings with different spatial contrasts (0–90% in 10% steps) and mean luminances (4–58 cd/m^2^ in 6 cd/m^2^ steps) were presented randomly and consecutively to the RF of a neuron in cat V1 (Fig. 1a). Each grating lasted for 20 ms (Fig. 1a). For each neuron, the size of the stimulus gratings was at least 3 times larger than its RF in diameter (far left panel of Fig. 1a), which was measured using sparse stimuli of bright and dark short bars that were presented by the reverse correlation method. The gratings had the preferred orientation and spatial frequency, as measured by the subspace reverse correlation method. Our previous study showed that a consecutive sequence of flashing gratings with luminances at different levels evoked significantly effective responses of V1 neurons to changes in stimulus luminance^14^. Although the stimuli we used were simpler than natural stimuli, the randomly and rapidly varying stimulus presentation simulated, to some extent, the simultaneous and rapid changes in luminance and contrast that occur during natural viewing^14^,^19^,^20^.

### Onset time of V1 response tuning to luminance changes was shorter than to contrast changes

**Figure 2:**
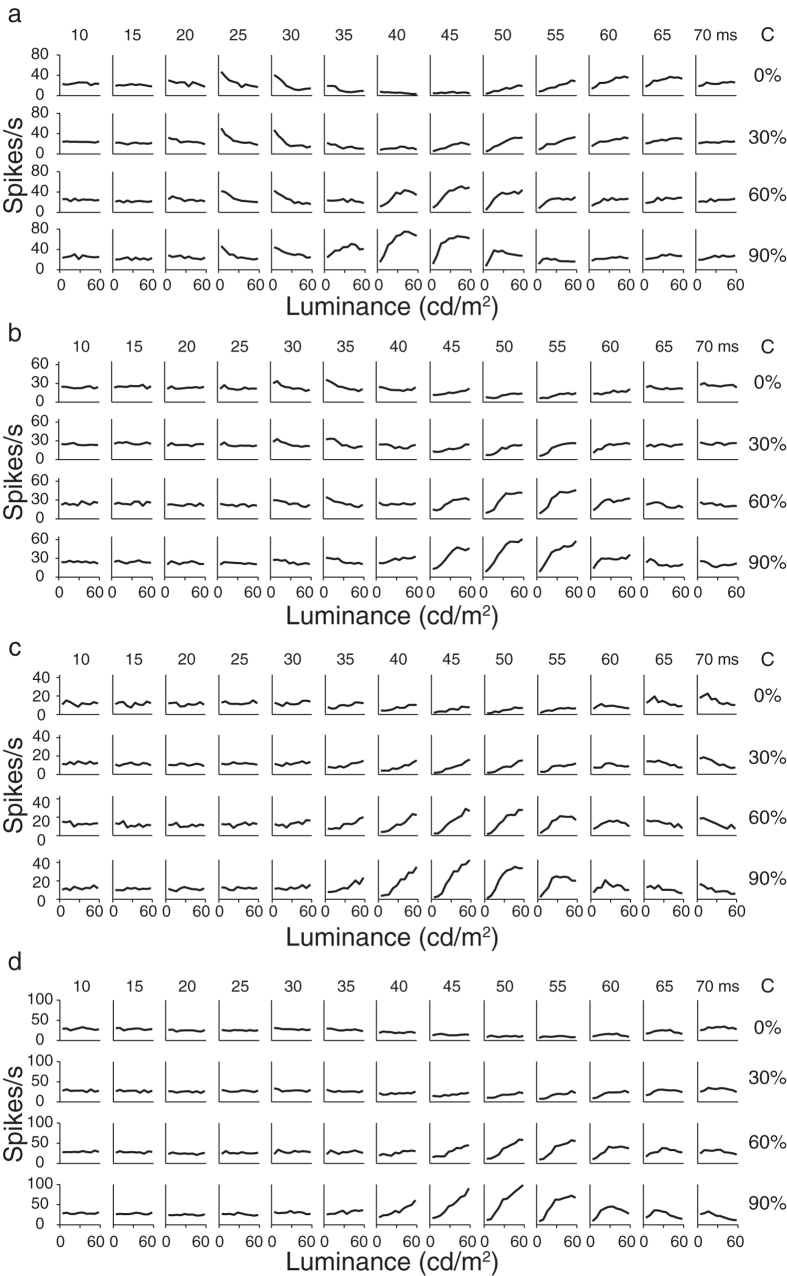
Time course of responses of V1 neurons to simultaneously changing luminance and contrast. (**a**–**d**) Luminance response functions of four neurons at four contrast levels (0%, 30%, 60%, and 90%) in 5 ms intervals. Neurons in (**a**–**c**) displayed the early responses (approximately 25–35 ms) to luminance changes of uniform stimuli (C = 0% contrast), while the neuron in (**d**) did not. The early responses of neurons in (**a**,**b**) preferred luminance decrements, while the neuron in (**c**) preferred luminance increments. The numerals at the top of each panel indicate the elapsed time after stimulus onset and the luminance response functions below were calculated at these time points. Neurons in (**a**,**b**,**d**) were complex cells, while the neuron in (**c**) was a simple cell. C: contrast (%).

Figure 1b shows the time course of the responses of a neuron to luminance and contrast changes under different luminance and contrast levels (shown for 4 luminance levels and 4 contrast levels for clarity). This cell had response peaks that occurred early after stimulus onset (short horizontal bar of Fig. 1b) under low luminance levels and across all contrast levels (left two columns of Fig. 1b). It had other response peaks that occurred late after stimulus onset under high luminance levels and high contrast levels (right two columns of Fig. 1b). It is worth noting that the early response peaks were similar across contrast levels. In contrast, the late response peaks occurred only with high luminance and contrast levels. To examine the effects of luminance and contrast changes on neuronal responses, we presented the responses of V1 neurons to simultaneously changing luminance and contrast as luminance response functions (LRFs) at different contrast levels. Figure 2 shows the LRFs of four neurons as examples, at four contrast levels (0%, 30%, 60% and 90%) and at temporal intervals of 5 ms. The neuron in Fig. 2a is the same neuron shown in Fig. 1b. This neuron had decreasing LRFs that were similar across different contrast levels and occurred early (25–30 ms of Fig. 2a), while had increasing LRFs in high contrast levels and that occurred late (40–45 ms of Fig. 2a). The magnitudes of the early decrement LRFs were dependent on luminance but were not dependent on contrast, while those of the late increment LRFs were affected by both luminance and contrast. The neuron in Fig. 2b was a typical example for the neurons that had decreasing LRFs in the early stage (30–35 ms), although with a relatively low magnitude. The neuron in Fig. 2c had increasing LRFs in the early stage (30–35 ms) that were similar across different contrast levels, while its late LRFs were similar to those of the two neurons of Fig. 2a,b. These data suggest that the early responses of the neurons were modulated by luminance, but not by contrast. This is compelling when comparing the LRFs to contrast gratings (bottom three rows of Fig. 2a–c) with those to uniform stimuli (0% contrast in the top rows of Fig. 2a–c). Responses of the three neurons in the late stage were scaled by luminance and contrast, suggesting that the late responses were modulated by both luminance and contrast. Many neurons had response patterns similar to these three neurons. However, some other neurons showed responses that were modulated by both contrast and luminance across the entire observation time (Fig. 2d). Most of the last type neurons did not respond to the uniform stimuli that had 0% contrast (first row of Fig. 2d). Their responses to luminance and contrast changes were late compared to the early responses of the former three neurons to luminance changes in Fig. 2a–c but were similar to the late responses of these three neurons to luminance and contrast changes. Different types of cells were recorded across all cortical layers of V1. We did not observe a clear difference in the distribution of these cells across cortical layers.

To quantitatively analyze this distinction, we extracted the onset time of responses of a neuron to luminance and contrast changes. In previous studies^21^,^27^, the maximal variance of responses of a neuron to different stimuli was taken as a measure of the response latency. When the maximal variance of responses occurs, it is the time when a neuron best tunes to different stimuli, which is called the peak or optimal latency. In these studies, stimuli were displayed continuously, without pause, and the presentation time for each stimulus was either short (several tens of milliseconds in the reverse correlation method) or long (320 ms). The short stimulus presentation in the current study was similar to that used by the above-cited studies.

**Figure 3:**
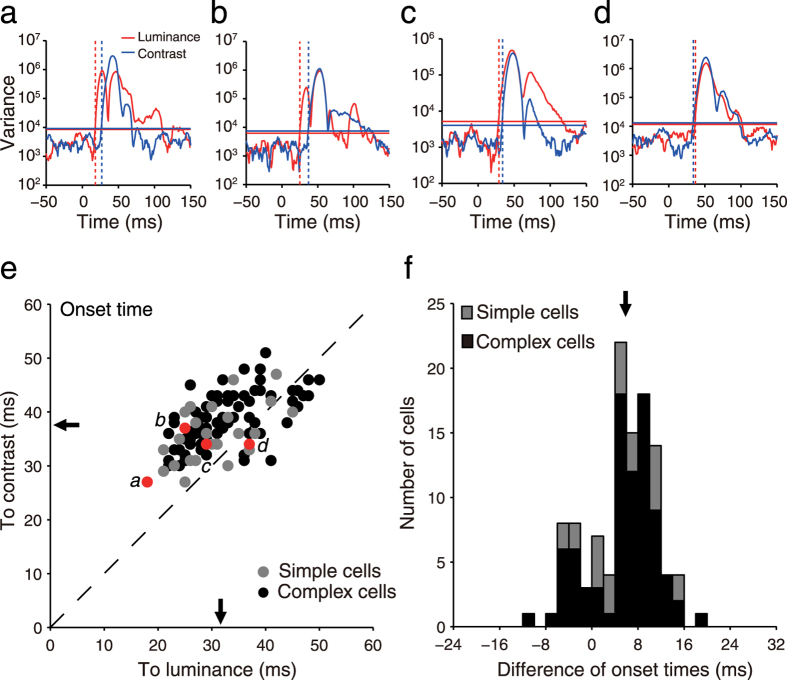
V1 neurons responded earlier to luminance changes than to contrast changes. (**a**–**d**) The time courses of response variances for the examples of neurons shown in Fig. 2a–d to luminance changes and contrast changes. The dashed vertical lines in each panel indicate the onset times of luminance responses (red) and contrast responses (blue). (**e**) Scatter plot of onset times of response tuning of 110 neurons to luminance changes and contrast changes. The broken line is the diagonal line. (**f**) Distribution of differences between onset times of response tuning of the neurons to contrast changes and those to luminance changes. Difference = contrast onset time — luminance onset time. Dark dots or bars in (**e**,**f**) represent complex cells, and gray dots or bars represent simple cells. Red dots (labels *a*, *b*, *c*, *d*) in (**e**) indicate the examples of neurons shown in Fig. 2a–d. Arrows indicate the mean values.

To relate responses of a neuron to two stimulus factors, luminance and contrast changes, a two-way analysis of variance (ANOVA) with replication^9^,^28^ was performed on responses of the neuron, with luminance as one factor and contrast as the other factor, at a millisecond interval ranging from 0 to 150 ms. We focused on the time when a neuron showed its first significantly differential responses to stimuli after stimulus onset (which we called the onset time of response tuning) instead of the peak latency, namely the time when showed the largest different responses (i.e., the maximal or peak variance of responses). This is because the onset time is the time when responses of a neuron first tune differentially to stimuli; in other words, when the stimuli first significantly affect responses of a V1 neuron. The onset time can be regarded as the time when useful information first reaches a neuron. The onset time of response tuning to luminance (or contrast) changes was defined by the following criteria. First, variance of responses to different stimuli after stimulus onset was beyond the mean + 5 SDs of variances of firing rates from −150 ms to 0 ms prior to stimulus onset calculated by the Equation (3) for stimulus luminance or Equation (4) for stimulus contrast. Second, two-way ANOVA indicated that at this time point, stimulus luminance (or contrast) significantly evoked differential responses (*P* < 0.0001 after Bonferroni correction for 450 multiple comparisons (i.e., 3 *F*-tests for each millisecond from 0 ms to 150 ms, α = 0.05/450 ≈ 0.0001), n = 800 repetitions). Third, the onset time was defined as the first one of five consecutive time points (in 1 ms step) when responses at all five of the time points met the previous two criteria. The neurons that met all three criteria above were included in the further analysis. Figure 3a–d shows the variance curves of responses of the four neurons in Fig. 2a–d and their onset times of luminance responses and contrast responses. For neurons in Fig. 2a–c, the onset times of responses to luminance changes were shorter than those to contrast changes (Figs 2a and 3a, luminance: 18 ms, contrast: 27 ms; Figs 2b and 3b, luminance: 25 ms, contrast: 37 ms; Figs 2c and 3c, luminance: 29 ms, contrast: 34 ms). For the neuron in Figs 2d and 3d, however, the onset time to luminance changes was similar to the onset time to contrast changes (luminance: 37 ms, contrast: 34 ms).

Of 110 neurons, 81% (89/110) had onset times to luminance changes that were shorter than their onset times to contrast changes and 71% (78/110) had onset times to luminance changes that were five or more milliseconds shorter than to contrast changes. That neuronal responses to luminance changes were faster than to contrast changes was further manifested by the data of the population of 110 neurons presented in Fig. 3e,f. The scatter plot between onset times to luminance changes and those to contrast changes (Fig. 3e) shows that most neurons (data points) were distributed above the diagonal line. The differences between onset times of these neurons to luminance changes and those to contrast changes were distributed largely toward positive values (Fig. 3f). On average, the onset time to luminance changes (31.7 ± 7 ms (mean ± SD hereafter for all data)) was significantly shorter than the onset time to contrast changes (37.5 ± 5 ms) by 5.8 ms ± 5.7 ms (*P* < 0.0001, paired *t*-test, n = 110). Next, we examined the time when the interaction between luminance and contrast changes began to significantly affect neuronal responses. The onset times were shorter for response tuning to luminance changes in 88% (92/105) of neurons and to contrast changes in 99% (104/105) of neurons than to the luminance-contrast interactions (for luminance-contrast interactions, 5 neurons did not meet the three criteria above). On average, onset times to luminance changes and to contrast changes were shorter than to luminance-contrast interactions (41.1 ± 5.8 ms) by 9.3 ms ± 6 ms (*P* < 0.0001, n = 105) and 3.9 ± 1.6 ms (*P* < 0.0001, paired *t*-test, n = 105), respectively. Becuase the variances of luminance-contrast interactions were much smaller than the response variances to luminance changes and to contrast changes, the effects of this interaction on neuronal responses were weak in comparison to the effects of luminance changes and of contrast changes.

Next, we examined whether the results were similar in simple cells and complex cells. A cell was classified as a simple or complex cell according to its modulation index (F_1_/F_0_) of responses to sinusoidal gratings drifting along its preferred direction^29^. Simple and complex cells had shorter onset times to luminance changes than to contrast changes by 5.3 ± 5.7 ms (*P* < 0.01, paired *t*-test, n = 25) and by 6 ± 5.8 ms (*P* < 0.0001, paired *t*-test, n = 85), respectively. The differences between onset time to contrast changes and to luminance changes were similar in simple and complex cells (*P* = 0.58, *t*-test). We observed that simple cells showed similar response profiles to luminance or contrast changes at the four spatial phase conditions of a stimulus grating (not shown). However, while the magnitudes of the early responses of simple cells to gratings at the four spatial positions (phases) were also similar, the magnitudes of their late responses to gratings at the four spatial positions were different. This suggests that the early responses of simple cells are not sensitive to contrast changes but that their late responses are sensitive to contrast changes because the modulation responses of simple cells to RF spatial phases are due to the fact that they have the maximal responses to contrast at the preferred spatial phase formed by ON and OFF subregions of their RFs. They display moderate responses to contrast at offset spatial phases and minimal responses to contrast at the opposite spatial phase^25^.

Our data showed that the onset time of response tuning of most V1 neurons, including both simple cells and complex cells, to luminance changes was significantly shorter than the onset time to contrast changes. These results indicate that most V1 neurons respond earlier to luminance changes than to contrast changes. Furthermore, that the onset time of responses to the interactions between luminance and contrast changes was longer than that of responses to luminance changes or to contrast changes also supports that the early responses to luminance changes are independent of contrast changes.

### Relationships between onset times of response tuning and RF properties

**Figure 4:**
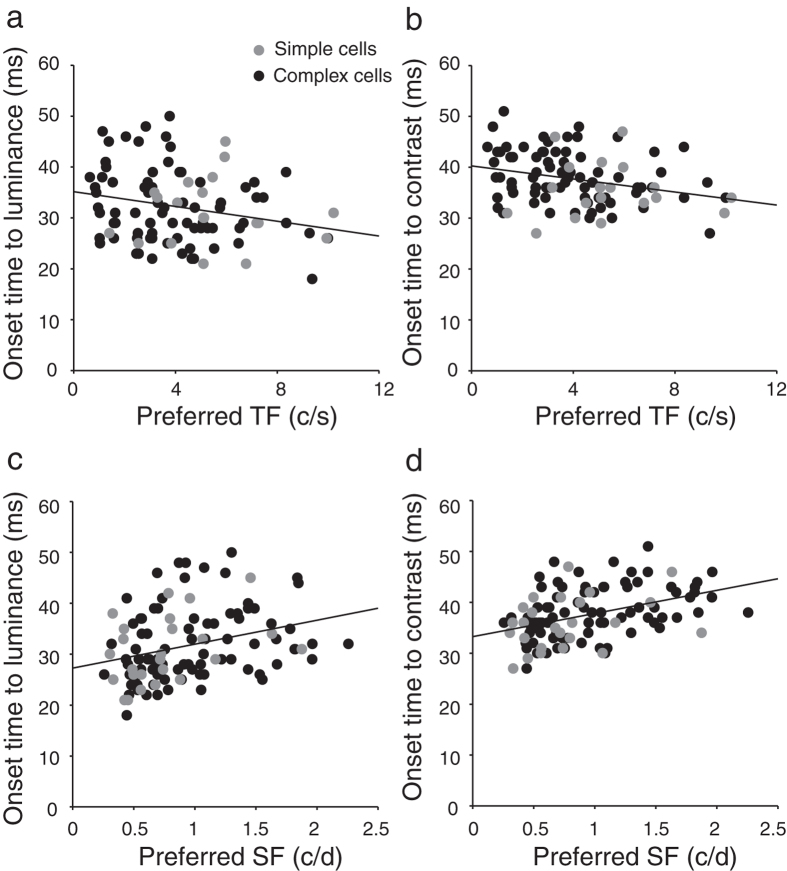
Correlation of response onset times of V1 neurons to luminance changes and contrast changes with their preferred temporal frequencies and spatial frequencies. (**a**,**b**) Scatter plots of onset times to luminance (**a**) and contrast changes (**b**) against the preferred temporal frequencies (TFs). The onset times of both luminance and contrast responses were significantly correlated with the preferred TFs (for luminance: *r* = −0.25, *P* = 0.02, n = 96; for contrast: *r* = −0.28, *P* < 0.01, n = 96; Pearson correlation test). (**c**,**d**) Scatter plots of onset times to luminance (**c**) and contrast changes (**d**) against the preferred spatial frequencies (SFs). The onset times of both luminance and contrast responses were significantly correlated with the preferred SFs (for luminance: *r* = 0.3, *P* < 0.01, n = 110; for contrast: *r* = 0.41, *P* < 0.001, Pearson correlation test, n = 110). Dark dots represent complex cells and gray dots represent simple cells. The lines were the linear regression lines. c/s: cycles/second. c/d: cycles/degree.

**Figure 5:**
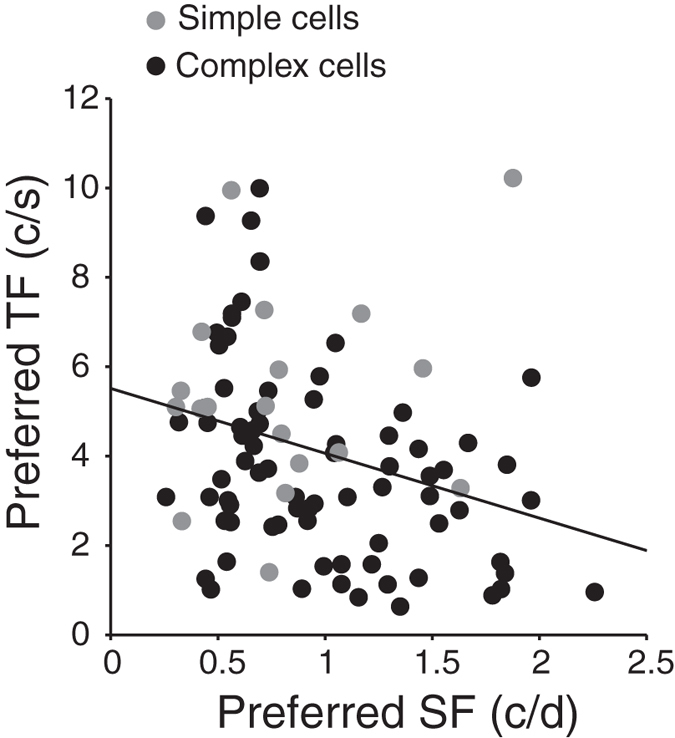
Correlation between the preferred temporal frequencies and spatial frequencies of V1 neurons. A Pearson correlation test indicated that the preferred temporal frequencies (TFs) and spatial frequencies (SFs) of V1 neurons were significantly correlated (*r* = −0.3, *P* < 0.01, n = 96). For other conventions, see Fig. 4.

We further examined whether the onset times of responses of neurons were related to the basic properties of their RFs. First, the onset times of neuronal luminance and contrast responses were plotted against the preferred temporal frequencies (TFs). Figure 4a,b show that the onset times to luminance and contrast changes both were significantly correlated with their preferred TFs (for luminance: *r* = −0.25, *P* = 0.02, n = 96; for contrast: *r* = −0.28, *P* < 0.01, Pearson correlation test, n = 96), suggesting that the neurons with a shorter onset time of response tuning tended to prefer a higher TF. Second, the onset times to luminance and contrast changes were also correlated to the preferred spatial frequencies (SFs) (for luminance: *r* = 0.3, *P* < 0.01, n = 110; for contrast: *r* = 0.41, *P* < 0.001, Pearson correlation test, n = 110; Fig. 4c,d), suggesting that the neurons with a shorter onset time tended to prefer a lower SF. Note that all of the neurons from which we obtained the preferred SFs were tested in the main experiment for luminance responses and contrast responses; however, some of these neurons had the preferred TFs beyond the range of TFs that we could test, and so we could not determine their preferred TFs. For these reasons, there was a difference in the number of cells in the analyses of onset time vs SF and of onset time vs TF. In addition, it is possible that TF and SF were themselves correlated. Figure 5 shows that TF was negatively correlated with SF (*r* = −0.3, *P* < 0.01, n = 96). Third, the spatiotemporal organization of the ON and OFF sub-regions of neuronal RFs was evaluated by the spatial and temporal overlap indices used in our previous studies^10^. The overlap indices ranged from negative values through zero to 1, which correspond to ON and OFF sub-regions that are totally segregated with a gap, or abutted against each other, or completely overlapping in a spatial or temporal dimension, respectively. No significant relationship was observed for the onset times of luminance and contrast responses of neurons with the spatial overlap indices of their RFs (for luminance: *P* = 0.28, n = 82; for contrast: *P* = 0.85, n = 82) or with the temporal overlap indices (for luminance: *P* = 0.14, n = 82; for contrast: *P* = 0.13, Pearson correlation test, n = 82).

## Discussion

Our study shows that response tuning of V1 neurons to luminance changes begins earlier than previously thought. When simultaneous changes in luminance and contrast stimuli were presented, responses of most V1 neurons to luminance changes preceded their responses to contrast changes. The early responses before the onset time of contrast responses were modulated by luminance and were independent of contrast, while the late responses were modulated by both luminance and contrast. Consistent with these findings, the neurons having shorter onset times of response tuning to luminance changes and contrast changes preferred higher TFs and lower SFs. These results were similar in simple cells and complex cells.

We also show that V1 neurons that have a lower preferred SF tend to prefer a higher TF. This is consistent with the previous studies^30^,^31^. The correlations between the onset times of neuronal luminance responses and contrast responses with their preferred SFs and TFs might reflect the neuronal connections that process luminance, contrast, SF, and TF, in which case it would be reasonable for the neurons that prefer higher TFs tend to respond earlier to luminance and contrast signals. That the early responses of the neurons preferring low SFs are sensitive to luminance changes (Fig. 4c) is in accord with the fact that luminance is the extremely low spatial frequency component of visual images. Since luminance is the most fundamental type of visual information, it is also understandable that luminance responses precede contrast responses.

It has been shown that the relationship between the stimulus luminance (or contrast) and neuronal firing rates is normally logarithmic. Most V1 neurons are of the logarithmic relationship. In the previous studies^12^,^32^, however, the presentation time of luminance or contrast stimuli was long, up to seconds, and also there was an interval between the two stimuli. In contrast, our stimulus presentation was short and continuous, without pause. In this case, the relationship between the stimulus contrast and the neuronal firing rate is relatively more linear, as is shown in our previous study where the presentation time was 40 ms^27^. For luminance, many V1 neurons also displayed this linear relationship in the current study (Fig. 2). It is possible that contrast responses are relatively more linear than luminance responses for stimulation of short duration. Although some neurons indeed had a logarithmic relationship in response to our short presentation of luminance stimuli, their stimulus-response relationship was relatively more linear than that for a long presentation. In consideration of these effects of both luminance and contrast, we applied linear parameters for luminance and contrast stimuli in 20 ms presentations in the current study. Thus, the linearly spaced parameters of luminance and contrast stimuli used in the current study were unlikely to have influenced our results. Although we did gamma correction for the CRT monitor, the grating stimuli with the mean luminance of 4 cd/m^2^ and 10 cd/m^2^ at the contrast of 10% were a little distorted in comparing with ideal sinusoidal curves. At the mean luminance of 4 cd/m^2^ and at contrast of 10% (the lowest mean luminance level tested in this study), four luminance values were available for generating the stimulus, and at the mean luminance of 10 cd/m^2^ and at contrast of 10%, six luminance values were available. Other stimuli could be generated by using more than eight luminance values. These stimuli (4 cd/m^2^ and 10 cd/m^2^ at low contrast of 10%), however, was a small number in the 10 × 10 stimuli we applied. In considering that we concerned the mean luminance of a grating stimulus rather than the absolute luminance at a local place, the few distorted stimuli at the low luminance levels might not affect our main results. Moreover, neuronal responses to low contrast (10%) stimuli were very weak when there were many stimuli at high contrast levels in the set of stimuli, the few distorted stimuli at the low contrast level unlikely affected the main results in the study if any effects. To confirm this, we reanalyzed the data of Fig. 3 by deleting the responses to all the stimuli at the low contrast of 10% (including the stimuli having the luminance of 4 cd/m^2^ and 10 cd/m^2^). This analysis gave almost the same results as those presented in Fig. 3. On average, the onset time to luminance changes was 32 ± 7.2 ms which was similar to 31.7 ± 7 ms of Fig. 3e (*P* = 0.07, paired *t*-test, n = 110), and that to contrast changes was 37.6 ± 5 ms which was also similar to 37.5 ± 5 ms of Fig. 3e (*P* = 0.13, paired *t*-test, n = 110). Thus, the effects of the distortion of the grating stimuli at low luminance and low contrast on neuronal responses were almost neglectable. Additionally, the method used an 11 ms of boxcar to smooth response PSTH in the study introduces a bias in measuring the onset time (<5 ms) of responses. If the noises during responses of a neuron to luminance changes and contrast changes were stable, the bias would be similar in estimating the onset time of luminance responses and that of contrast responses.

The early contrast-independent luminance responses were not observed in a previous study on responses to simultaneously changed contrast and luminance^13^. One possible reason for the discrepancy is the difference in the visual stimulus. We presented sinusoidal gratings steadily flashed for 20 ms without an inter-stimulus interval, while Geisler *et al.* presented sinusoidal gratings that drifted for 200 ms followed by an inter-stimulus interval blank of the mean luminance for 200 ms. Another possibility is that the excitatory responses to luminance are weak^10^ and that the instantaneous changes in luminance evoke strong cortical inhibition^17^. In Geisler *et al.*’s study^13^, the early weak responses to luminance were hard to detected due to the low or absent spontaneous rates of V1 neurons^33^ when stimuli were presented with an inter-stimulus blank interval. Low spontaneous activity results in low background activity, which does not favor the detection of weak signals^34^. The consecutively and randomly flashing stimuli that we applied elevated the baseline and thereby enhanced the sensitivity of V1 neurons to weak signals^34^,^35^,^36^,^37^. Moreover, consistent with our current study, a previous intracellular *in vivo* study in tree shrew V1 showed the initial luminance responses to luminance step gratings that were invariant for all orientations^17^.

An important question is where the early contrast-independent luminance responses come from. Our results show that simple cells in V1 exhibited the early luminance responses (Fig. 3e,f). It has been shown that simple cells reside mainly in input layer 4 and upper layer 6 and receive direct convergent inputs from the ON and OFF relay cells of LGN in the cat^2^,^38^,^39^. Thus, the early luminance responses of simple cells are not likely to be generated by intra-cortical circuits in V1 or by feedback inputs from the higher order areas of the visual cortex, and they must therefore originate from LGN inputs. In addition, a minority of complex cells in the superficial layers and layer 5 also receive direct inputs from cat LGN^39^. For these complex cells, the early luminance responses may also originate from LGN inputs. Moreover, our results show that the neurons with shorter response onset times to luminance and contrast stimuli prefer higher TFs and lower SFs (Fig. 4), which is consistent with our previous finding that the V1 neurons that respond to uniform luminance stimuli and grating contrast stimuli prefer higher TFs and lower SFs than those neurons that respond only to grating contrast stimuli^10^,^14^. It has been shown that in the retino-geniculate pathway of cats, Y cells have shorter latencies and prefer higher TFs and lower SFs than X cells^40^,^41^,^42^,^43^,^44^. Furthermore, Y cells in the cat LGN respond 10–15 ms earlier than X cells^40^. Combining these results with our current result, i.e., that the responses of V1 cells to luminance changes are faster than the responses to contrast changes, it suggests the possibility that the early luminance responses derive mainly from the subcortical Y cells.

Our study indicates that for most V1 neurons, luminance responses are faster than contrast responses. The early responses represent luminance, while the late responses represent both luminance and contrast, suggesting that the temporal course of visual information processing is from luminance to contrast signals in V1, with the processing for luminance lasting longer. The previous studies have shown that responses to low spatial frequency precede those to high spatial frequency in LGN and V1 cells^24^,^27^,^45^. Uniform stimuli and mean luminance are the extremely low spatial frequency components of visual scenes. Thus, the information stream from luminance to contrast is consistent with that from low to high spatial frequencies. The temporal course of spatial frequency tuning is thought to reflect “coarse-to-fine” visual processing, which has also been described in RF size tuning in V1 simple cells^46^, in orientation tuning in V1 cells^23^,^47^, in 2D shape selectivity tuning in V2 cells^48^, in shape and face processing in the inferior temporal cells^49^, and in shape and face processing in human cortex as revealed by fMRI and ERP^50^. Therefore, the initial processing for local luminance signals forms the basis of the subsequent processing for other features, such as local contrast, spatial frequency, and orientation.

Regarding the representation of a surface with uniform luminance in a large visual area, a process of slow scale-dependent neural filling-in is thought to be involved. Responses of a portion of V1 cells to the interior of a uniform surface are slower than their responses to the oriented contrast border of the same uniform surface^11^. In our study, some neurons had the onset time of responses to luminance stimuli that were longer than that of their responses to contrast stimuli (dots below the diagonal line of Fig. 3e), and it might be these neurons that show slow responses to a uniform surface. The slow surface responses are hypothesized to be induced by contrast at the oriented border of a uniform surface. Then, in V1, where the uniform surface is represented, the slow surface responses diffuse from the border to the interior region of the uniform surface^11^,^12^,^51^,^52^,^53^. These slowly propagating responses can provide a neural substrate for the perceptual filling-in of large uniform luminance, the induced brightness of the Craik–O’Brien–Cornsweet effect, and simultaneous brightness contrast^12^,^51^,^52^,^53^,^54^. The responses from the contrast border to the surface luminance represent a “fine-to-coarse” process. Thus, “coarse-to-fine” and “fine-to-coarse” processing are not mutually exclusive because they are different parts of the same overall neural process that occurs over time (coarse-to-fine-to-coarse)^55^. Our current study suggests that the early responses to luminance are the initial and fundamental step of the “coarse-to-fine-to-coarse” process by which we perceive visual objects.

## Methods

### Physiological Preparations

Eleven normal adult cats (2–3 kg) were prepared for extracellular recording^10^. The initial anesthesia of an animal was induced by injecting ketamine (20–30 mg/kg, i.m.), dexamethasone, and atropine (i.m.). Under deep anesthesia with a combination of propofol and sufentanil, craniotomy surgery centered at the Horsley-Clarke coordinates P 2.5 mm and L 2.5 mm was performed. The anesthesia and paralysis of the animal were maintained with an infusion of sufentanil (0.15–0.22 μg/kg/hr, i.v.), propofol (1.8–2.2 mg/kg/hr, i.v.), and gallamine triethiodide (10 mg/kg/hr, i.v.) in a physiological solution of 5% glucose during the recording. End-tidal CO_2_ and body temperature were kept at 3.5–4.2% and approximately 38 °C, respectively. The eyeballs of the animal were covered with contact lenses of sufficient power and 3 mm artificial pupils to prevent them from drying and to focus his eyes on a CRT monitor at a distance of 57 cm. To avoid light scatter effects from other sources and reflections in the experiment room, the space around the animal and the monitor was covered by black boards that allowed the animal’s eyes to face straight forward only to the monitor. The extracellular action potentials of V1 cells were recorded with a glass-coated tungsten electrode of 1–3 MΩ impedance, amplified and filtered with a TDT amplifier, and collected with a data acquisition system (TDT, Inc, Florida) at a 12 kHz sample rate. The RFs of the cells located in the region of area V1 that represented the central region of the visual field. Usually, neurons in V1 (area 17) and V2 (area 18) in cats can be distinguished by their preferred SFs^25^,^56^. Single units were further identified from the recorded spike trains, while off line, with a TDT OpenSorter. All animal care and experimental procedures conformed to the guidelines of The National Institutes of Health (USA) and were approved by the Institutional Animal Care and Usage Committee (IACUC) of The Institute of Biophysics, Chinese Academy of Sciences.

### Visual Stimuli

Stimuli were generated by C# code and presented on a cathode-ray tube monitor (Iiyama HM204DT A) at a resolution of 800 × 600 pixels and with a refresh rate of 100 Hz. The monitor subtended approximately 40 × 30° of visual angles in a dark room at 0.1 cd/m^2^ luminance, as measured by a ColorCAL colorimeter (CRS, Ltd). The gamma correction was applied to the CRT monitor by using its inverse gamma function to calculate the input RGB indices for a stimulus.

After isolating the action potentials of a neuron, we measured the approximate size and location of the RF, and the preferred orientation, SF, and TF of the recorded neuron for the dominant eye. The other eye was covered. The parameters were qualitatively explored by varying stimuli controlled by a computer mouse along these dimensions. After the preliminary tests, we quantitatively measured the selectivity of the neuron for orientations (0°–165° in 15° steps) and SFs (0.1–2.3 cycles/degree, depending on the preferred SF of the neuron) by a subspace reverse correlation method^21^,^23^,^25^. The classical RF of the neuron was then quantitatively measured using statically flashing white and dark short bars (1.5° × 0.5°) presented by the conventional reverse correlation method^21^,^22^,^25^,^57^. Preference for TFs (0.3125–10 cycles/second) was measured with sinusoidal gratings that drifted along the preferred direction. The gratings had 100% contrast and the preferred SF and orientation.

Visual stimuli of circular sinusoidal gratings with different spatial contrasts (0–90% in 10% steps) and mean luminances (4–58 cd/m^2^ in 6 cd/m^2^ steps) were consecutively and randomly presented to the RF of the neuron being recorded to investigate dynamic responses to simultaneously changing luminance and contrast (Fig. 1). Each of the 100 grating stimuli was presented for 20 ms. Each grating had four spatial phases (0, π/2, π, and 3π/2). After a random sequence of the 100 stimuli × 4 phases was finished, a new sequence of the stimuli was presented for a total of 200 sequences. Each of 100 combinations of 10 stimulus luminances × 10 stimulus contrasts was thus presented for 800 repetitions (4 × 200). The border of a grating stimulus was blurred by a smooth change in luminance from the stimulus to the background (Fig. 1a). The non-blurred region of grating stimulus was at least 3 times larger than the RF in diameter. The width of the blurred border region equaled that of non-blurred interior region of the gratings^14^. Gratings had the preferred orientation and SF of the neuron being tested. The stationary sinusoidal gratings were described by the following equations:


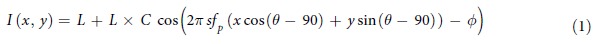


where 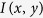 defined the light intensity at position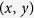, 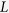 was the mean luminance and 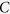 was the contrast (Michelson contrast) of the gratings, 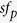 was the preferred spatial frequency (cycles/degree), 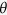 was the preferred orientation (degree), and 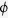 was spatial phase. The contrast of the gratings was Michelson contrast:


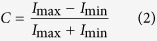


where 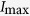 and 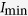 were the maximal and minimal light intensity of the sinusoidal gratings, respectively.

## Data analyses

### PSTHs and noise level estimation

For a given stimulus, spike times (1 ms resolution) from 800 stimulus repetitions were aligned to the stimulus onset time and were analyzed over the period from 150 ms before to 200 ms after stimulus onset. PSTHs were obtained over this period of 350 ms^27^,^58^,^59^. Responses (PSTHs) were smoothed with a (boxcar) window of 11 ms in steps of 1 ms. The spike rate at the time point x ms was averaged from those during from -5 ms before x ms to 5 ms after x ms. The variance of responses of a cell was calculated from its smoothed PSTHs to different stimuli in steps of 1 ms. To estimate the noise level of responses of a cell, the mean of the variances of responses from −150 to 0 ms before stimulus onset was taken as the baseline noise level. The mean + 5 SDs of the variances was taken as the criterion. If the variance of responses of a cell to different stimuli after stimulus onset exceeded this criterion, the cell was regarded to have significant responses and was further analyzed.

### Two-way ANOVA

To determine the onset time of response tuning that was evoked by luminance changes and contrast changes, we performed two-way ANOVA with replication for responses of a neuron, with luminance as one factor and contrast as the other factor, at each millisecond from 0 ms to 150 ms. Responses in each trial (repetition) were smoothed by a window of 11 ms, and thus the response at each time point was the mean spike rate of 11 ms that was centered at that time point. The sum of the squares (SS) of the deviations from the mean were calculated by following equations:


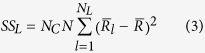



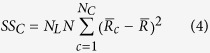



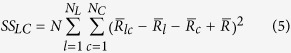



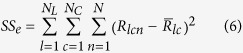


where 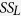, 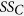, 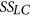, and 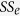 were the SS of responses to luminance changes, contrast changes, luminance-contrast interaction, and within-group noise, respectively. 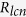 was response to a stimulus with luminance level 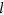 and contrast level 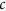 at the 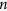-th repetition. 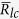, 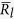, 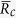, and 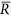 were the mean of responses to a stimulus with luminance 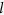 and contrast 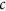 (across repetitions), the mean of responses to stimuli with luminance 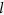 (across contrast levels and repetitions), the mean of responses to stimuli with contrast 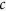 (across luminance levels and repetitions), and the mean of responses to all the stimuli (across luminance levels, contrast levels, and repetitions), respectively. 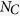 and 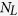 were the numbers of luminance levels and contrast levels, respectively, and both equal to 10. 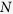 was the number of repetitions, and equals to 800. The variances were calculated by following equations:


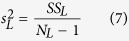



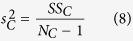



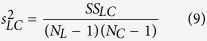



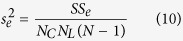


where 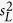, 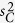, 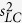, and 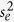 were the variances of responses to luminance changes, contrast changes, luminance-contrast interaction, and within-group noise, respectively. The effects of luminance changes, contrast changes, and luminance-contrast interaction were tested by *F*-tests:


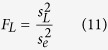



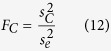



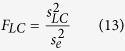


where 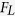, 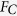, and 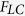 were the *F* values of responses to luminance changes, contrast changes, and luminance-contrast interaction, respectively. The onset time of response tuning to luminance changes (or contrast changes or luminance-contrast interaction, respectively) was defined by three criteria: 1. The 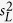 (or 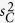 or 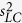) of responses after stimulus onset were larger than the mean + 5 SDs of 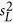 (or 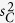
or 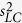) of firing rates during −150 to 0 ms before stimulus onset; 2. The *P* value of an *F*-test (luminance: 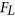; contrast: 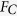; luminance-contrast interaction: 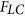) was smaller than 0.0001 after Bonferroni correction for 450 multiple comparisons (3 *F*-tests for each millisecond from 0 ms to 150 ms, α = 0.05/450 ≈ 0.0001, n = 800 repetitions); and 3. The onset time was the first time point of five consecutive time points when responses to luminance changes (or contrast changes or luminance-contrast interaction) at all five of the time points met the previous two criteria. The neurons that met all three criteria above were included in the analysis of onset time.

#### RF properties of neurons

To discriminate between simple cells and complex cells, the ratio of the amplitude of the first harmonic and the mean response rate (F_1_/F_0_) of responses to drifting gratings were calculated. Cells were classified as simple cells if F_1_/F_0_ > 1; otherwise, they were classified as complex cells^29^.

RF spatial and temporal structures were analyzed as described in our previous studies^10^. Briefly, the spatial and temporal overlap indices of ON and OFF sub-regions that were measured using the conventional reverse correlation method^21^,^22^,^25^,^57^ were calculated.

The TF tuning curves were fitted by a log-Gaussian function^60^:


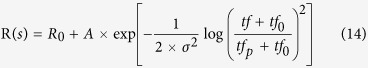


where 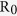, 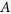, 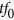, 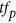, and 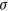 were parameters, 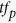 was the preferred TF, and 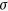 was the width of the tuning curve. Neurons with extremely high TFs (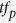, cycles/second) that were beyond what we could test were not included in the analyses. The SF tuning curves were also fitted by the log-Gaussian function.

## Additional Information

**How to cite this article**: Wang, W.-L. *et al.* V1 neurons respond to luminance changes faster than contrast changes. *Sci. Rep.* **5**, 17173; doi: 10.1038/srep17173 (2015).
